# Assessment of foraminal decompression following discoplasty using a combination of ex vivo testing and numerical tools

**DOI:** 10.1038/s41598-023-27552-0

**Published:** 2023-02-25

**Authors:** Chloé Techens, Ferenc Bereczki, Sara Montanari, Aron Lazary, Luca Cristofolini, Peter Endre Eltes

**Affiliations:** 1 Department of Industrial Engineering, School of Engineering and Architecture, Alma Mater Studiorum - Università di Bologna, Bologna, Italy; 2 In Silico Biomechanics Laboratory, National Center for Spinal Disorders, Királyhágó St. 1-3, Budapest, 1126 Hungary; 3 Department of Orthopaedics, Department of Spine Surgery, Semmelweis University, Üllői St. 78/b, Budapest 1082, Hungary; 4 School of PhD Studies, Semmelweis University, Budapest, Hungary

**Keywords:** Biomedical engineering, Musculoskeletal system

## Abstract

Percutaneous Cement Discoplasty (PCD) is a minimally invasive surgical technique to treat degenerated intervertebral discs. When the disc is severely degenerated, the vacuum observed in place of the nucleus pulposus can be filled with bone cement to restore the disc height, open the foramen space, and relieve pain. This study aimed to evaluate the foramen geometry change due to PCD, in the loaded spine. Cadaveric spines (n = 25) were tested in flexion and extension while Digital Image Correlation (DIC) measured displacements and deformations. Tests were performed on simulated pre-operative condition (nucleotomy) and after PCD. Registering DIC images and the 3D specimen geometry from CT scans, a 3D model of the specimens aligned in the experimental pose was obtained for nucleotomy and PCD. Foramen space volume was geometrically measured for both conditions. The volume of cement injected was measured to explore correlation with the change of foramen space. PCD induced a significant overall foraminal decompression in both flexion (foramen space increased by 835 ± 1289 mm^3^, *p* = 0.001) and extension (1205 ± 1106 mm^3^, *p* < 0.001), confirming that the expected improvements of PCD show also during spine motion. Furthermore, in extension when the foramen is the most challenged, the impact of PCD on the foramen correlated with the injected cement volume.

## Introduction

Advanced degeneration of the intervertebral disc deeply affects the biomechanics and structure of the spine. The loss of disc height associated to the degeneration leads to the reduction of the neural foramen and a compression of the nerves inducing low back pain in the patients. Percutaneous Cement Discoplasty (PCD) is a minimally invasive surgical technique developed as an alternative for patients not suitable to lumbar interbody fusion surgery due to advanced age or comorbidities^1^. During surgery, the degenerated disc is filled with injected acrylic bone cement to create a spacer between the vertebrae and indirectly decompress the nerves by restoring the disc height and the neuroforaminal space^1^. Postoperatively, PCD significantly decreased pain level in patients of various studies and improved their quality of life^2^. With only 4% of cement leakages as most common complication, PCD is a promising alternative minimally invasive treatment to interbody fusion for polymorbid patients. In particular, PCD is recommended for those patients whose intervertebral discs exhibit a vacuum phenomenon as the result of severe degeneration, as such void is suitable for being filled with bone cement^1^.

The application of PCD in the lumbar spine has been mostly studied clinically. First, the effects of PCD have been assessed in term of disc height restoration on CT scan images, pain relief and quality of life improvement^3^. Then, the biomechanical behaviour of the treated spine as well as its alignment under loading have been studied using animal ex vivo model, to gain a better understanding of the consequences of discoplasty in flexion, extension and lateral bending^4^. Additionally, an in silico approach was developed by Eltes et al*.* for the quantitative investigation of the indirect changes induced by PCD inside the spinal canal and the neuroforamen in patients^5^. Because the procedure is based on the indirect decompression of the neuroforamen (e.g. without resecting the compressing tissue), the investigation of the foramen volume was preferred compared to other methods to overcome the limitations of the classical 2D method. It was also more relevant due to the foramen irregular shapes that can be found in patients with advanced degeneration. This patient-specific method used clinical CT scan-based images of the patient in prone/supine position.

In spine research, in vivo assessments are always constrained by the need of using only non-invasive and safe acquisition tools. These tools also often fail to control the imposed conditions, and cannot measure some physical quantities (e.g. force, stress, relative motions) in the internal spine structure. Indeed, medical imaging often requires the patient to be static, whereas tracking the spine motion is performed externally (e.g. with stereophotogrammetry) and does not allow direct insight of the internal tissue.

This study aimed to assess the changes of foramen characteristics following PCD in the most concerning motions. It was hypothesized that (i) PCD would significantly affect the foramen space in flexion and in extension, and (ii) that the volume of cement injected would correlate with the foramen change. For that, we developed a workflow combining the ex vivo data of the spine segments in loaded configurations and the in silico tools to investigate the foramen geometry to bridge clinical and experimental approaches and widen the investigation of PCD. In particular, we aimed to measure the impact of discoplasty on the shape and size of the foramen in complex motions.

## Methods

This study is a combination of ex vivo investigation of spine biomechanics^4^ and in silico image analysis similar to a clinical approach^5^. Ex vivo data acquisition is extensively described in Supplementary Information S1.

### Acquisition of ex vivo data

Lumbar functional spinal units (FSU, n = 25) were transected from 15 donors whose death was unrelated to spine condition (68 ± 13 years old). This study was performed in line with the principles of the Declaration of Helsinki, the National Regulations, and approved by the Local Ethics Committees (Bioethics Committee of the University of Bologna, Prot. 76497, 1 June 2018). The informed consent was obtained from all subjects and/or their legal guardian(s)/donors. The cadaveric spines were obtained through two institutions: an international donation program (International Institute for the Advancement of Medicine) and the hospital of the NCSD after ethical approval of both entities.

**Figure 1:**
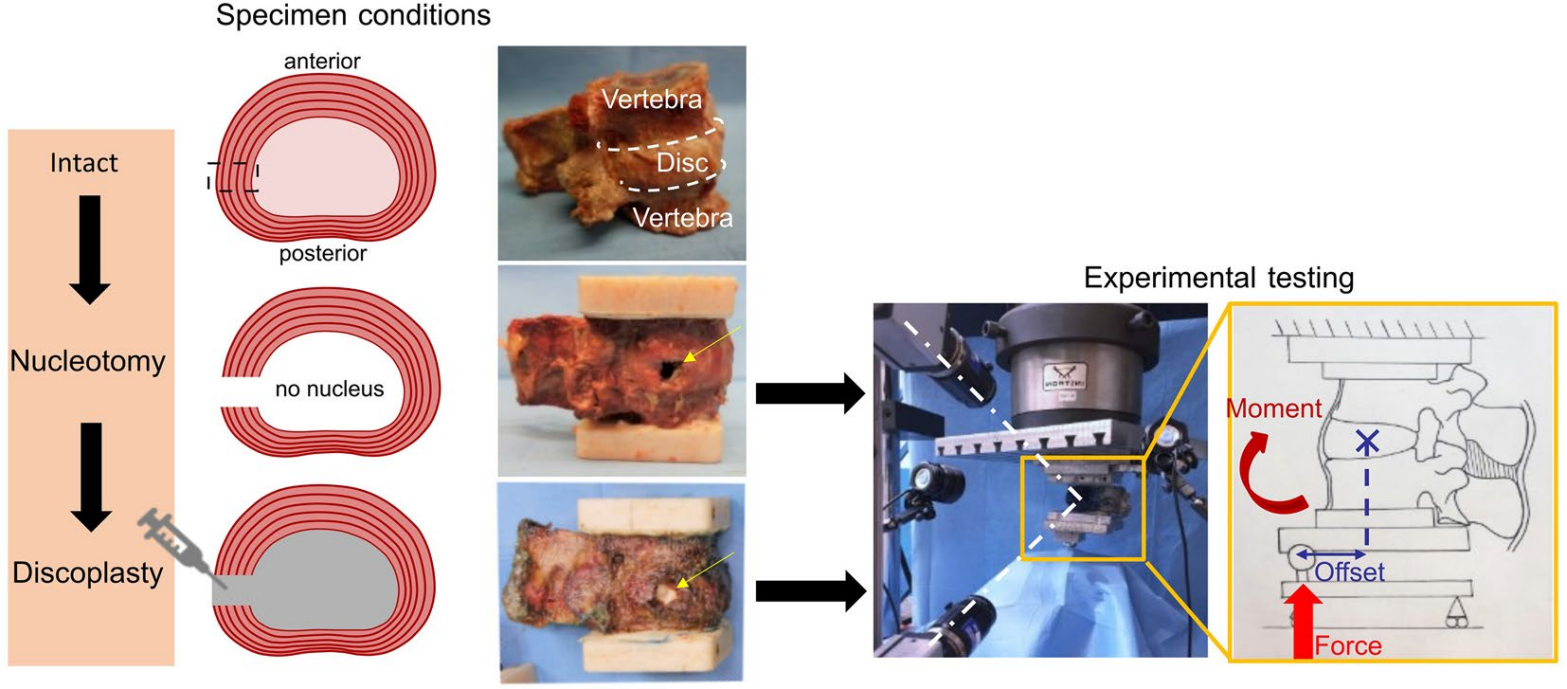
Preparation and test of one specimen in flexion. The discs were tested in nucleotomy and discoplasty. The eccentric load was applied to create the bending moment (in flexion here) while DIC system recorded the specimen position (white dot lines).

The specimens were cleaned without damaging ligaments and facet joints. PCD is a surgery which aims to indirectly decompress the foramen compared to the preoperative state. No quantification of the decompression relative to healthy condition is given as an objective in surgical planning. Only comparative with the preoperative state is performed to evaluate the benefits of the surgery. Similarly, the in vitro models provided data on the preoperative and postoperative conditions. Because vacuum is rarely observed among cadaveric spines but is a requirement of PCD, degeneration of intervertebral discs was modelled by manually removing the nucleus pulposus and the inner layer of annulus through a square opening performed on the lateral side of the discs^4^. The degeneration state of the specimens was not evaluated since only the vacuum was relevant for the study. Cement discoplasty was performed by injecting acrylic cement (Tecres, Sommacampagna, Italy) through the opening (Fig. 1). Between the preparation and the testing phases, specimens were stored at − 28 °C, and were thawed in physiological solution at room temperature prior to each test session^6^.

Specimens were tested in both nucleotomy and simulated discoplasty conditions, to assess the impact of the cement injected on the kinetics. The specimens were mechanically tested in flexion and extension using an uniaxial testing machine (Mod. 8032, Instron, UK). One pot was rigidly fixed to the top of the testing machine and the other was loaded through a spherical joint moving along a low-friction rail (Fig. 1). A force of 50% of the Body Weight (BW), representing the upper body above the lumbar vertebra, was applied with an anterior (posterior) offset, generating a combination of compression and flexion (extension) (Supplementary Table 1). The offsets were defined from the centre of the disc and were scaled on each specimen according to the antero-posterior length of the disc: an offset of 35% of such length was used to generate flexion, and 70% of the length to generate extension. Some specimens exhibited a large mobility after nucleotomy, then, to avoid specimen damage, a reduced load was applied (Supplementary Table 1).

Each test consisted of 6 loading cycles: a 1.0 s loading ramp; a 0.3 s hold at maximum load, then the unloading. Three cycles are sufficient for minimizing the effect of the viscous component in the response in another study^4^. Each 6-cycle test was repeated five times. Before the tests, each specimen was pre-conditioned applying the test load as a sinusoid at 0.5 Hz for 20 cycles. The specimens were tested in nucleotomy and cement discoplasty conditions for both directions of loading. During the test, a digital image correlation (DIC) system monitored the lateral side of the specimens and measured the displacements of the surface. The system was previously validated^7^, and DIC parameters such as facet size and grid spacing were optimized to minimize the result errors prior to test acquisition^4^. The accuracy on the position detected by the DIC system was of the order of 2 µm (1/20 to 1/50 of the pixel size is typical of DIC). During testing, three specimens were damaged, in a way which could only affect flexion results. These tests were excluded from the results as a precaution, thus leaving n = 94 tests available.

### Post processing of the DIC data

Throughout the testing, the DIC measurements (n = 94) provided the coordinates of the specimens’ surface points corresponding to the correlated area on the surface of the two vertebrae and intervertebral disc. The frame at peak load were selected for each test. The 3D correlated area was converted into a point cloud using an iterative-closest-point algorithm (‘icpregister’, Matlab, R2019b, MathWorks, Inc., Natick, MA, USA) and imported into MeshLab1.3.2 (http://www.meshlab.net) (Fig. S1.1). The normals of the mesh vertices were estimated considering a neighbourhood of 100 points. Then, the specimen surface was reconstructed from the point cloud with normals using the Ball Pivoting Algorithm^8^. This algorithm was iteratively applied with ball radius of 0.8 mm, 1.0 mm, 1.10 mm (clustering radius: 20% of ball radius, angle threshold: 90°) in order to include the maximum of vertices and to fill the smaller holes resulting from the lack of correlation. An ultimate loop with a ball radius of 1.25 mm was performed unless it led to the superposition of new mesh triangles with former ones. The clustering radius of this loop was reduced to 10% in order to avoid that issue^8^. The reconstructed surface was stored as a STL file.

In order to identify the vertebral surfaces only, for each specimen the reconstructed DIC surfaces corresponding to the four tests (nucleotomy and discoplasty, each loaded in flexion and extension) were imported into a modelling software ‘3-matic’ (Mimics InnovationSuite v23.0, Materialise, Leuven, Belgium). Using a ‘brush’ tool, the parts of the DIC surface corresponding to the vertebra were manually selected and copied separately as new parts (n = 188).

### CT scan data acquisition and processing

The specimens were scanned with a clinical computed tomography (CT) scanner (Aquilion ONE, Toshiba; with 220 mA, 120 kV, 0.3 mm slice thickness, 0.214 mm pixel size), after discoplasty before testing.

**Figure 2:**
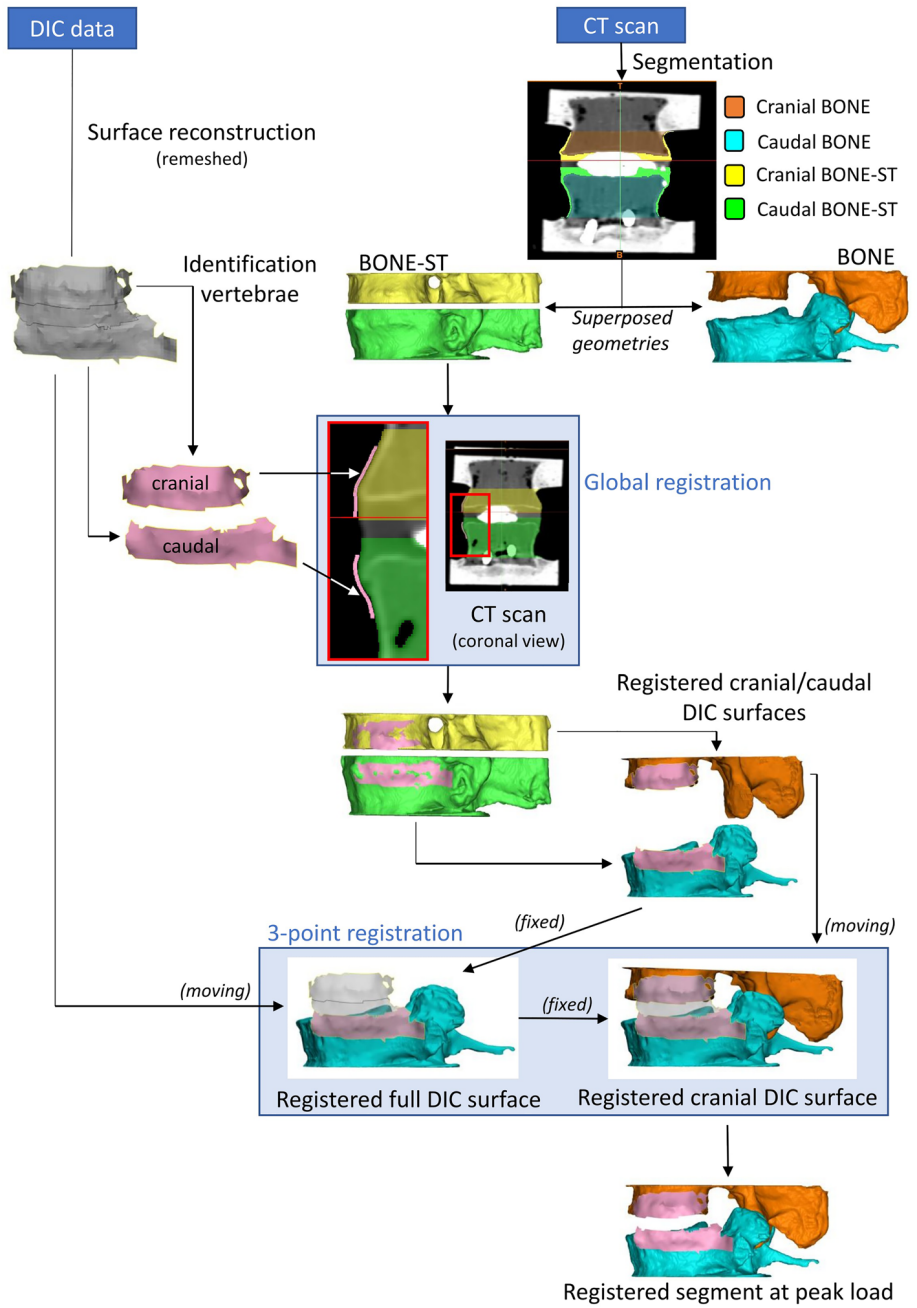
Registration process from the CT pose to the experimental pose. Registration of DIC meshes onto the corresponding BONE-ST segmented masks was first performed. After registration, the position of DIC in the space mask remained unchanged whether BONE-ST or BONE part is considered due to the masks’ superposition. The vertebrae were then aligned in the experimental pose using registration between the DIC meshes.

The caudal and cranial vertebrae (called BONE masks) of each specimen were segmented into Mimics® image analysis software (Mimics Innovation Suite v23.0, Materialise, Leuven, Belgium) from the DICOM images using thresholding and some manual edits to separate the vertebrae from the injected cement. A second segmentation of the same vertebrae was performed: in order to match the surface recorded with DIC, both vertebrae with the remaining soft tissue around were segmented with threshold > 700 HU (BONE-ST masks) (Fig. 2).

### Registration of the DIC and CT data

The rigid registration relied on the assumption that the two vertebrae behave as rigid bodies displacing in space (especially the cranial one, which was not constrained) while the connecting soft tissue were deformed. In order to associate the DIC surface meshes and the segmented geometries from the CT scan, the first step consisted in registering the DIC surface of each vertebra on the corresponding BONE-ST masks (Fig. 2). For that, DIC surface meshes (n = 188) of cranial and caudal vertebrae were imported into Mimics for the four tests (nucleotomy and discoplasty, each loaded in flexion and extension). A first manual alignment of caudal and cranial DIC meshes (moving element of the registration) on the corresponding caudal and cranial BONE-ST masks (fixed element of the registration) was performed in the sagittal, frontal, and transverse views using the ‘reposition’ tool. Then each DIC surface mesh (moving element of the registration) was globally registered on the corresponding BONE-ST mask (fixed element of the registration) using the ‘STL registration’ tool.

**Figure 3:**
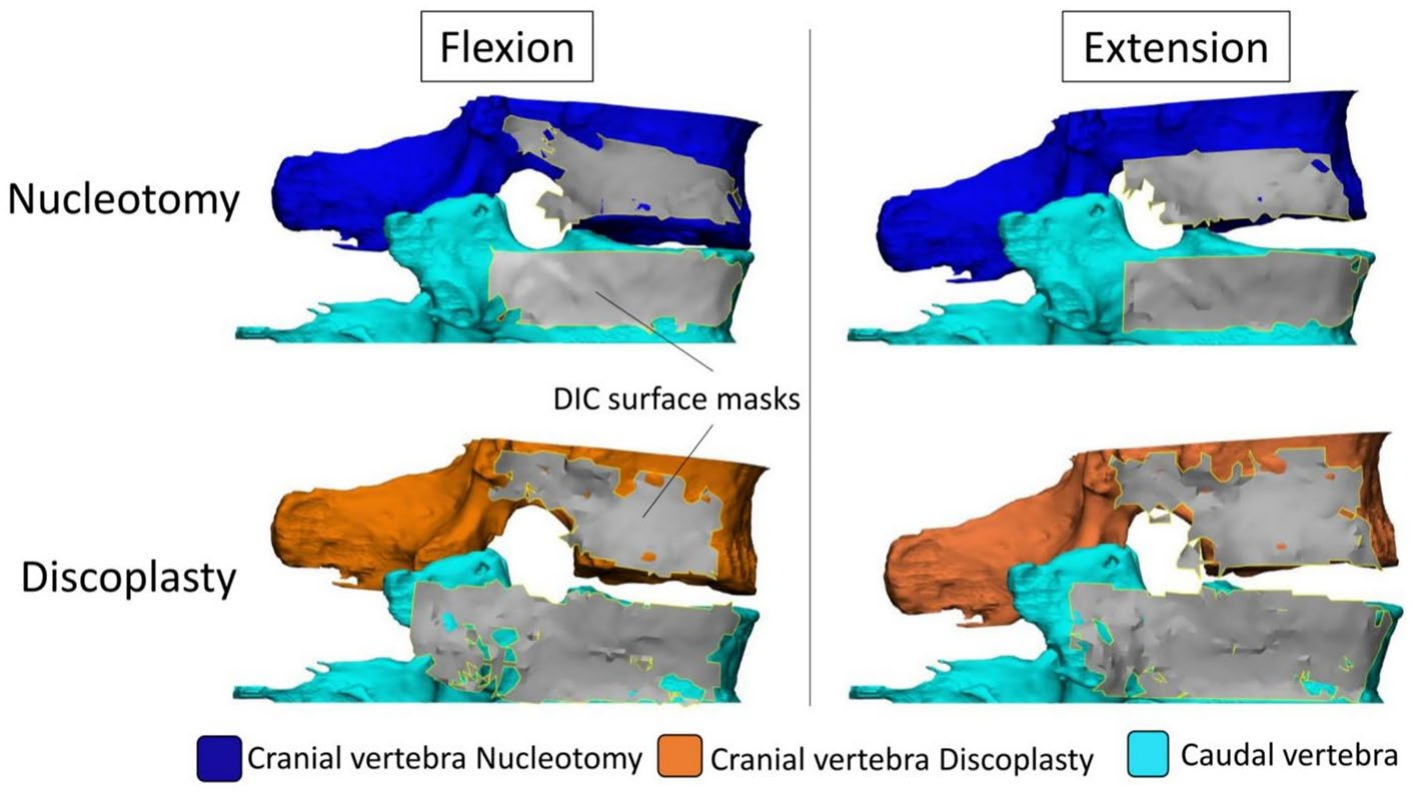
Representative specimen with DIC surface masks (grey) registered for the four test configurations. The complete set of specimens is available from the figshare database (accession number 10.6084/m9.figshare.19196237).

In order to move the 3D geometry of the cranial and caudal vertebrae from the CT pose to the experimental pose, the segmented BONE masks were first converted into STL parts. Then, the cranial and caudal BONE parts and the cranial and caudal registered DIC meshes were copied into ‘3-matic’ where the DIC full surface mesh (two vertebrae + disc) was already imported. In order to align the BONE parts in the experimental pose, two registrations were performed using the ‘3 point registration’ tool (for each specimen, this process was repeated for the four tests: nucleotomy/discoplasty, each loaded flexion and extension) (Fig. 3):

- The DIC full surface mesh was registered to the caudal registered DIC mesh. The meshes being identical, the selected points were identical vertices in both meshes and the point registration was perfect.
- Similarly, the cranial registered DIC mesh was superposed to the DIC full surface mesh. In order to match the position recorded with the DIC, the cranial BONE part conjointly moved with its registered cranial DIC mesh. This resulted into the cranial and caudal BONE parts in the same position as the vertebral bodies at full load during the ex vivo tests.

### Measurement of the neuroforaminal 3D geometry

**Figure 4:**
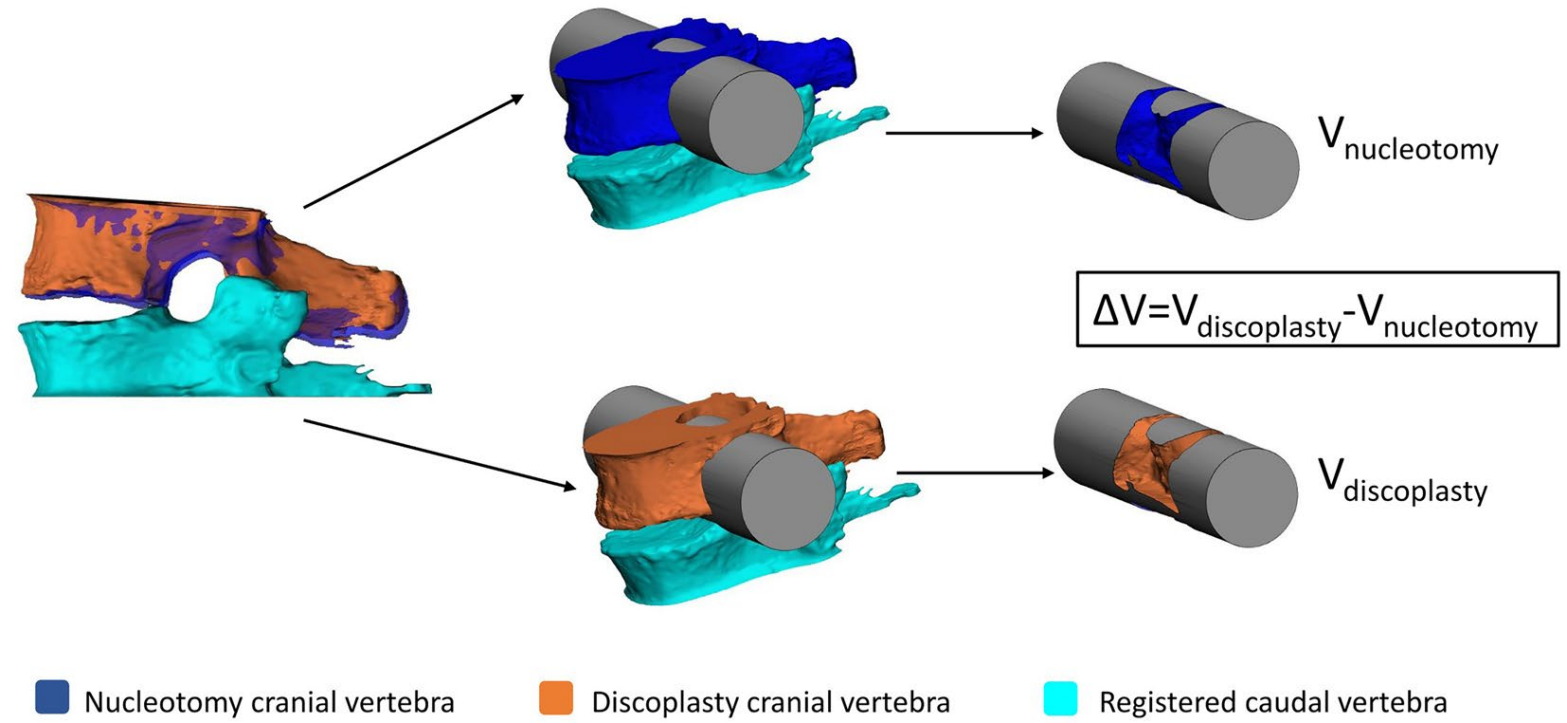
Foramen volumetric measurement method. Adapted from^5^ under the CC BY license (http://creativecommons.org/licenses/by/4.0/).

Once the BONE parts were correctly aligned, the neuroforamen change due to discoplasty was measured using a method developed previously for this purpose^5^. Indeed, most of the studies use 2D parameters (disc height, foramen height/diameter, foramen cross section, etc.) to assess the foramen, assuming that a pitch point results from a reduction of the foramen in a parasagittal plane. In case of advanced degeneration, spines exhibit more complex foramen geometries (stenosis, osteophytes, etc.) and this volumetric method presented the advantage of considering the 3D geometry of the spinal canal, providing the accurate description of its dimensions and evaluating the indirect decompression effect. A measurement cylinder was drawn and aligned in the virtual transverse axis of the two neuroforamen in ‘3-matic’ software. The cylinder was 90 mm long while its radius was adjusted for each specimen and motion (flexion/extension) in order to entirely fill the foramen of both nucleotomy and discoplasty conditions. The intersection of the vertebrae with the cylinder was subtracted from the cylinder, providing the free foramen volume V_nucleotomy_ and V_discoplasty_ (Fig. 4). The volumetric change (ΔV = V_discoplasty _− V_nucleotomy_) of the foramen between nucleotomy and discoplasty was computed for flexion and for extension.

### Evaluation of the measurement quality

The whole workflow was reproduced by 2 operators (O_1_ and O_2_) who repeated it 2 times (T_1_ and T_2_) each. The inter-operator repeatability of the segmentation was measured with Dice Similarity Index (DSI). Only the segmentation of the BONE masks was evaluated since it might directly impact the foramen volume. The BONE-ST mask segmentation did not involve subjective operator intervention and was not evaluated. The quality of the global registration between the DIC meshes and the segmented BONE-ST masks was measured with root-mean-square error (RMSE), indicating the average distance error between the 2 elements. To evaluate the repeatability of the entire procedure, the Hausdorff Distance (HD) of the two registrations of the same DIC surface mesh (O_1_T_1_ vs O_1_T_2_, O_2_T_1_ vs O_2_T_2_, O_1_T_1_ vs O_2_T_1_, and O_1_T_2_ vs O_2_T_2_) was measured with the MeshLab1.3.2 software (http://www.meshlab.net) Metro tool^9^. As also the quality of the DIC-correlated surfaces was suspected to affect the overall registration quality, its features such as DIC surface area and DIC surface roughness, were investigated in depth: this analysis showed that the registration errors did not depend on the quality of the DIC surfaces (See Supplementary Information S2 for details).

### Statistical analysis

All statistical analyses were performed with SPSS Statistics 25.0 (IBM Corp., Armonk, NY, USA) using nonparametric tests. Inter-rater (O_1_ vs O_2_) reliability was determined by Intraclass Correlation Coefficient (ICC) estimates and their 95% confident intervals (CI) were calculated based on a mean rating (k = 4), absolute-agreement, 2-way mixed-effects model. Intra-rater (O_1_T_1_ vs O_1_T_2_, and O_2_T_1_ vs O_2_T_2_) reliability was determined by ICC estimates and their 95% CI were calculated based on a single measurement, absolute-agreement, 2-way mixed-effects model. ICC was applied for the foramen space measurements. The statistical significance in the change of foramen volume from nucleotomy to discoplasty was assessed by a paired Wilcoxon’s test. The relationship between the mean volumetric change (ΔV) and the cement volume was investigated with Spearman’s rank correlation. P-value smaller than 0.05 were considered significant.

## Results

### Evaluation of the registration procedure

The DSI for the segmentation of the vertebral bodies only (BONE masks) was 0.99 ± 0.01 (mean ± s.d.), indicating a high reproducibility of the procedure and its operator independence.

The RMSE was automatically retrieved for each vertebra, disc state and loading configuration to evaluate the matching of the DIC surface with the corresponding BONE-ST mask. Overall, the two operators O_1_ and O_2_ exhibited an RMSE of 0.27 mm ± 0.10 mm and 0.24 mm ± 0.08 mm respectively, showing of the high repeatability of the method.

**Figure 5:**
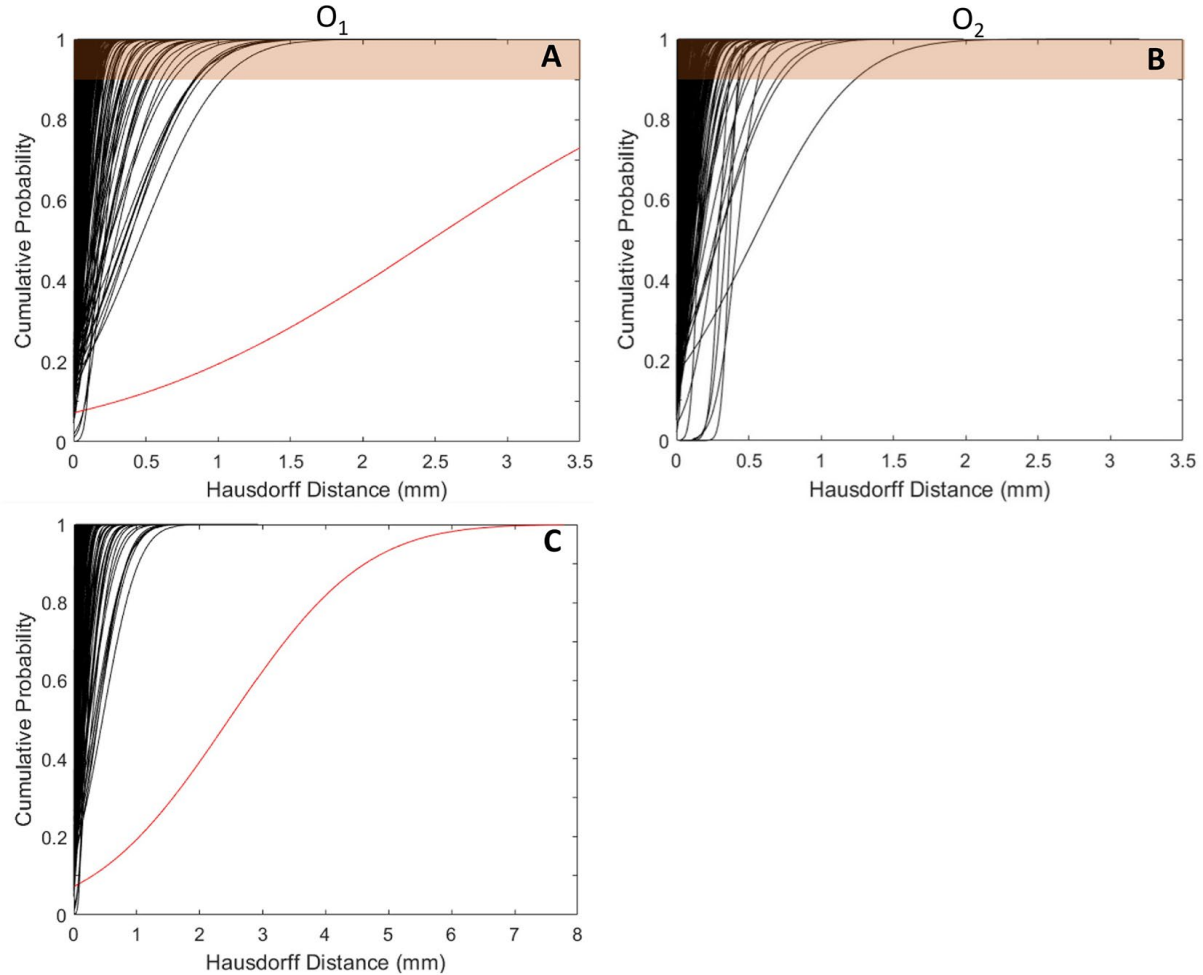
HD cumulative probability of operators O1 (**A**) and O2 (**B**) for each DIC mask registration (188 curves in total corresponding to 25 specimens, with 2 vertebrae each, tested in 2 disc conditions and 2 loading configurations, with exclusion of 3 tests in flexion). One DIC mask registration showed very large HD values compared to the others (**C**).

The reproducibility of the registration was evaluated computing the Hausdorff Distance (HD) for each DIC mask between its registration at two times (T_1_, T_2_), and by two operators (O_1_, O_2_). The HD values were computed for each node of the mask; the minimum, mean, maximum, and RMS HD values over each mask were extracted (Supplementary Table 2). Both operators exhibited low HD values (mean ± s.d.) of 0.09 ± 0.19 mm (O_1_) and 0.07 ± 0.07 mm (O_2_) which demonstrated of the intra-operator similarity of the registrations. Similarly, the mean HD values measured between the registrations of each operator were 0.17 ± 0.22 mm (T_1_) and 0.16 ± 0.20 mm (T_2_), and showed the low influence of the operator on the registration results. To more deeply assess the repeatability of the registration method, cumulative probability plots of the HD values of each mask were created for and between both operators (Fig. 5A,B). Almost all the masks (~ 99%) had an HD value < 1 mm at 90% of the mesh nodes. One mask for O_1_ (Fig. 5C) exhibited a mean HD exceeding the others by 8 times the standard deviation (2.46 mm). The corresponding registration was considered not reliable enough to be included in the foramen measurements, and the causes of this poor registration were investigated (Supplementary Information S2).

### Foramen decompression induced by discoplasty

To test the reproducibility of the method, the measurements of the foramen volume after nucleotomy and discoplasty were performed by two operators repeated twice. Inter-rater reliability for O_1_ mean (T_1_,T_2_) versus O_2_ mean (T_1_,T_2_) was ICC = 0.898 (CI 95%, Lower bound = 0.828, Upper bound = 0.937). Intra-rater reliability for O_1_T_1_ versus O_1_T_2_ was ICC = 0.987 (CI 95%, Lower bound = 0.980, Upper bound = 0.991) and for O_2_T_1_ versus O_2_T_2_ ICC = 0.994 (CI 95%, Lower bound = 0.992, Upper bound = 0.996). These results confirmed of the high repeatability and reproducibility of the foramen volume measurements.

**Figure 6:**
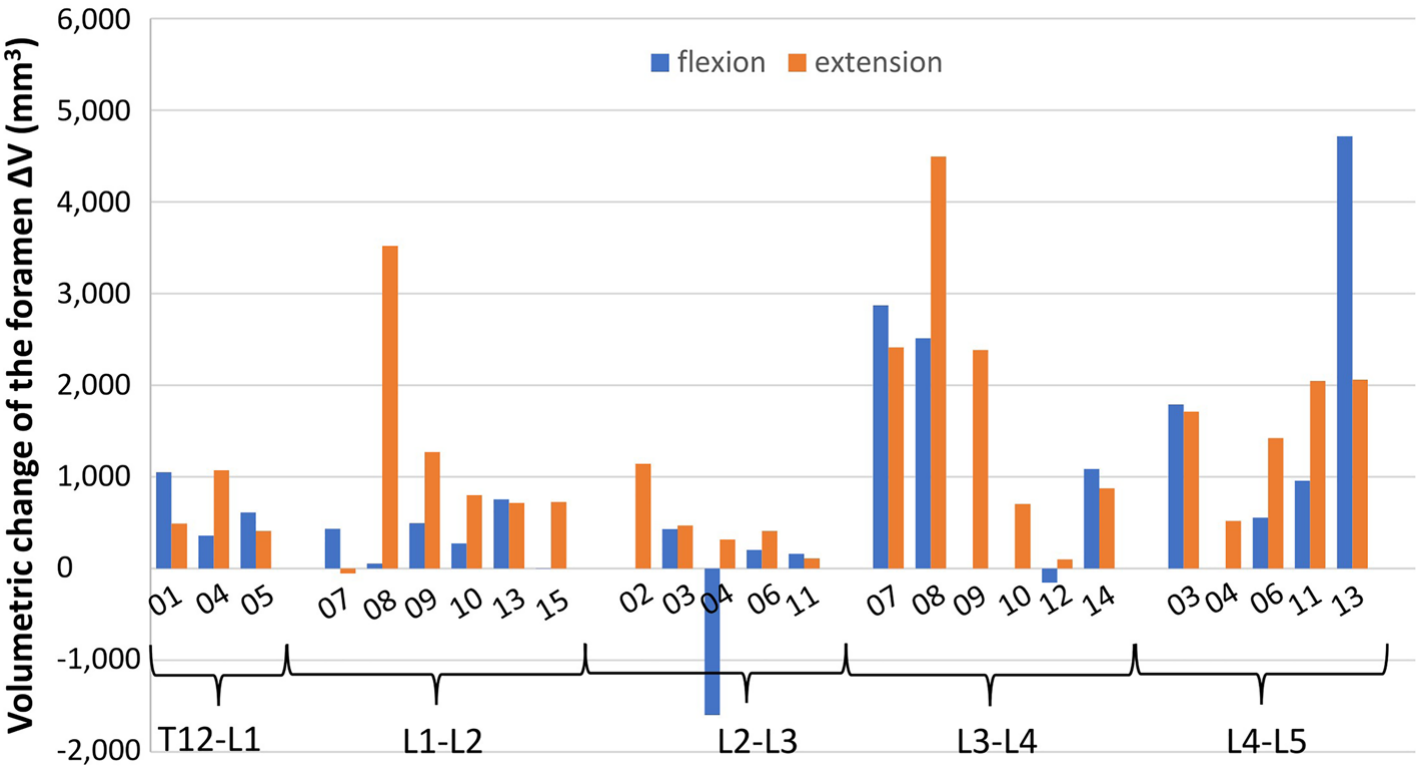
Change of the foramen volume after discoplasty in flexion and extension for every specimen. The mean was computed across all operator measurements and all repetitions (O1T1, O1T2, O2T1, O2T2).

The volume of the foramen was compared after nucleotomy (V_nucleotomy_) and after discoplasty (V_discoplasty_) for flexion and extension. A large variability in term of foramen change (ΔV = V_discoplasty_ − V_nucleotomy_) was observed among the specimens for both operators (Fig. 6). The volume increased by 835 ± 1289 mm^3^ (V_nucleotomy_ vs V_discoplasty_, *p* = 0.001, paired Wilcoxon’s test) in flexion, and 1205 ± 1106 mm^3^ (*p* < 0.001) in extension, Supplementary Table 3). Results of the decompression measured by O_1_ and O_2_ are reported in Supplementary Tables 4 and 5. The specimens were sorted by spine levels, resulting in a larger mean volumetric change in the levels L3-L4 and L4-L5 for both flexion and extension but no significant difference was found between spine levels (Fig. 6, Supplementary Table 6).

### Correlation between cement volume and foraminal decompression

**Figure 7:**
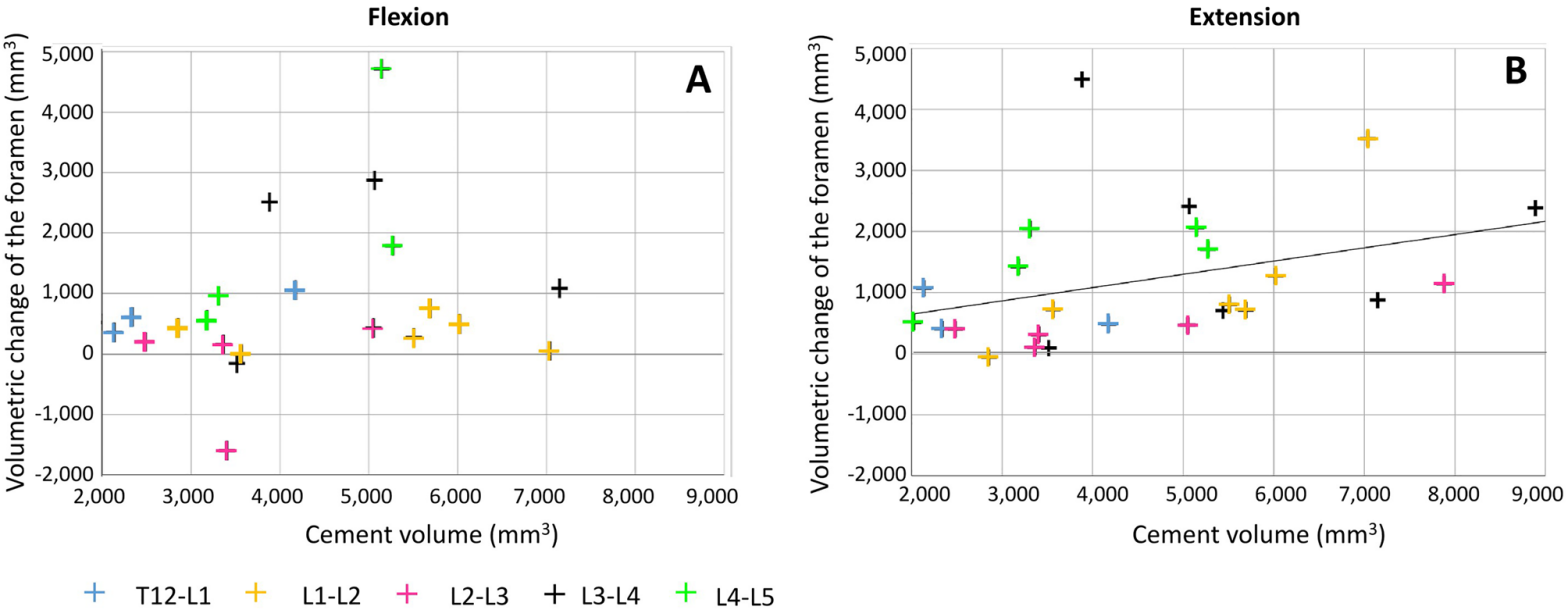
Correlation between the volume of cement injected and the foramen volumetric change induced by PCD: in flexion (**A**), and extension (**B**). Linear regression is plotted for all data.

The cement volume measured for every specimen was 4575 ± 1854 mm^3^ (mean ± s.d.). In flexion, a weak positive correlation was observed between the volume of injected cement and the ΔV of the spinal canal (Spearman’s test, ρ = 0.273, *p* = 0.232, not significant). A significant moderate positive correlation was observed in extension (ρ = 0.470, *p* = 0.018) (Fig. 7).

## Discussion

In this paper, an investigation of the changes in the foramen geometry was conducted on ex vivo data in order to estimate the impact of PCD when the spine is loaded in flexion and extension. The 3D vertebra geometries were obtained from CT scans of the ex vivo specimens and then aligned in the experimental bending pose using registration of DIC data. The alignments were repeated for the specimens in pre- and post-operative conditions allowing the measurement of the foramen volume change implied by PCD.

**Figure 8:**
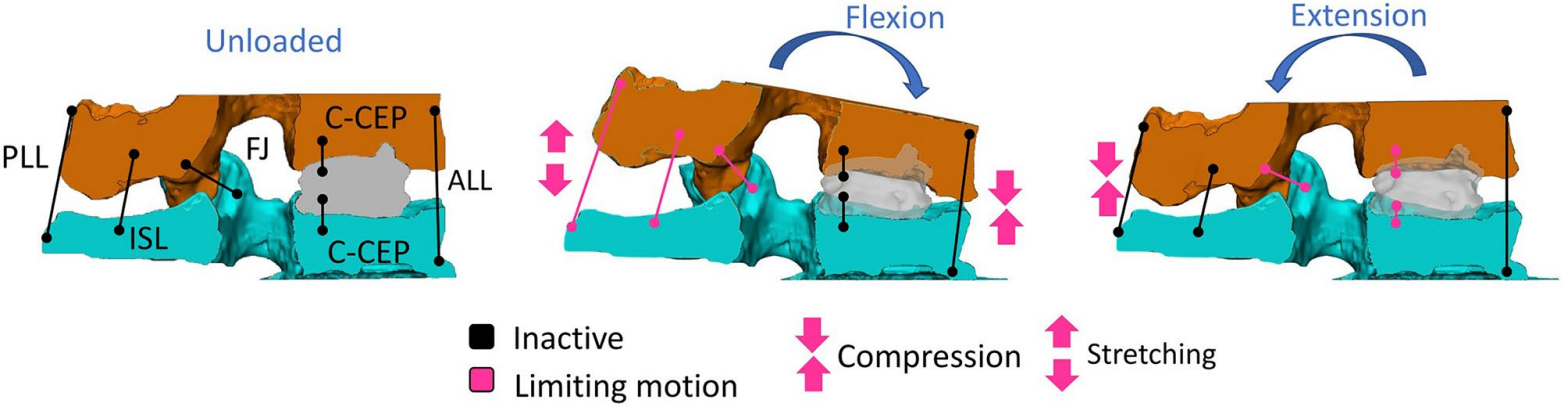
Influence of the cement mass (grey) in the specimen’s kinematics in flexion and extension. The main components (ligaments, contact surfaces, joints) are shown inactive (black) or active (pink) when they limit the motion, depending on the tested configuration. ALL: Anterior Longitudinal Ligaments, FJ: Facet Joints, ISL: Interspinous Ligament, C-CEP: Cement-Cartilaginous Endplate interfaces.

The segmentation and registration methods showed low intra- and inter-operator differences demonstrating of the repeatability and reproducibility of the workflow. In addition, registration of the DIC surface masks onto 3D vertebrae was proven to be precise, exhibiting a mean error in the range of the CT scan resolution. Thus, the combination of ex vivo experimentally acquired data and clinical images allowed registering the 3D geometries of the vertebrae in the actual pose under load, and to compare the condition after nucleotomy and after discoplasty. The foramen volumetric variations were investigated using a method previously developed on in vivo data which reported a decompression effect about 2295 ± 1181 mm^3^ in supine position^5^. Similarly, PCD significantly decompressed the foramen both in flexion and extension with values in the same order of magnitude despite the difference of spine loading. The volumetric change was smaller in flexion, probably because the cement mass is only indirectly involved in this direction of motion. Indeed, flexion was mainly constrained by the soft tissue (ligaments and facets) and limited by the anterior endplate contact (Fig. 8). The cement mass contributed to a larger stretch of the posterior ligaments and wider opening of the foramen. In extension the combined action of the injected cement and the posterior elements (facet joints) tended to constrain motion at an early stage compared to the nucleotomy condition, thus leaving more foramen space. All specimens showed an increase of the foramen volume, except one in flexion. This exception could be explained by the relative position of the cement mass within the disc and the loading application axis. As the cement was located most anteriorly in the nucleus space, where the axial load was applied, it limited the bending rotation of the vertebrae compared to the free rotation observed after nucleotomy, resulting in a decrease of the foramen volume. The volume of cement injected in vitro (Fig. 6) was 2–9 ml, in the range of 3–10 ml clinically reported^2^. Finally, larger volumes of cement were associated with larger increase of the foramen space in extension only, due to the direct involvement of cement for this direction of motion. Similar relation had been observed in vivo in supine position^5^.

To the authors’ knowledge, there are very few studies addressing the impact of PCD on the foramen space^3^. The method of 3D assessment of the foramen volumetric change using a cylinder was developed by Eltes et al*.*^5^ to provide an appropriate tool to evaluate the impact of PCD on the spine on living patients when the foramens exhibit a more complex geometry due to advanced degeneration. The study demonstrated that PCD increased the foraminal dimensions in neutral position and that patients’ pain reduction strongly and positively correlated with the foramen decompression. The geometrical change was also proven to have a strong positive correlation with the volume and surface area of injected cement. This could partially explain the larger change in foramen observed in vivo (2295 mm^3^ ± 1181, n = 16)^5^ than ex vivo since the volume of cement injected during patient surgery was in average larger than ex vivo. In Eltes et al*.*^5^, the impact of PCD on foraminal space was investigated on patient’s CT scans recorded in supine position. Because numerous repetitions of in vivo tomography to investigate different patient positions is time-consuming and can increase radiation exposure, in silico or ex vivo approaches are alternative methods allowing to easily vary the poses. Besides, for ex vivo investigation, the combination of DIC images with CT scans images proposes a cheaper and more versatile way to investigate the specimen geometry during loading compared to in situ CT imaging, in particular for long tests and large specimen cohorts.

However, our study also presented some limitations. Only 25 specimens were tested here: while this was sufficient to observe the general effect, a larger cohort would also allow to explore the dependence of the outcome on the spine level. The applied load was selected in order to preserve specimen integrity in absence of soft tissue and nucleus while creating realistic kinematics. However, it did not include a preload which could result in lower loads in the in vitro model than applied physiologically. FSUs were used to model the spine behaviour, differing from physiological anatomy. This could partially explain the reduction of one foramen volume found here, whereas no such case was reported after PCD^5^. Moreover, despite the high accuracy and precision showed by our method, the registration repeatability and replicability were found to be increased with large DIC mask surface areas. Although a narrow field of view is not systematically associated with poor registration, one of tests had to be excluded from our cohort for this reason. Thus, acquiring a larger portion of the specimen surface would provide more nodes in the mesh to register, and then a decreased error. The more cameras a DIC system does include, the larger the field of view is^10^. With such set-ups, the workflow of this study could be automatized using larger DIC masks.

Our study investigated the foramen volume at peak load in flexion and extension. The foramen changes during the loading phase were not investigated. Due to the limited knowledge on PCD biomechanics, it cannot be excluded that the foramen is also challenged during the loading phase. However, Eltes et al*.*^5^ found a strong correlation between the decompression volume and patients’ leg pain, suggesting peak load would be a critical scenario. Further dynamic investigations on the foramen would be needed to complete the assessment of the foramen changes.

## Conclusion

Following a first clinical investigation of the in vivo foraminal space after PCD^5^, this study aimed to develop a more detailed ex vivo analysis of the foramen geometrical changes induced by the surgery in simplified, yet representative loading configurations. For that, ex vivo biomechanical testing and CT imaging were combined to create the 3D geometry of the spine segments in preop- and postop- conditions. This work allowed to draw some conclusions corroborating the PCD benefits on the foramen space.

- PCD induced a significant decompression of the foraminal space under flexion and extension, showing the positive biomechanical effect of the surgery under loading.
- The increase of foramen space was positively correlated with the volume of injected cement in extension, confirming the clinical observations.
- This method could be applied for the assessment of the spine geometry under various other loading conditions as long as the DIC-acquired field of view is large enough.

## Supplementary Information

Supplementary Information.

## Data Availability

The data file is available from the figshare database (accession DOI number 10.6084/m9.figshare.19196237). The rest of data can be found in the supplementary information.

## References

[1] Varga PP, Jakab G, Bors IB, Lazary A, Szövérfi Z (2015). Experiences with PMMA cement as a stand-alone intervertebral spacer. Orthopade.

[2] Techens C, Eltes PE, Lazary A, Cristofolini L (2022). Critical review of the state-of-the-art on lumbar percutaneous cement discoplasty. Front. Surg..

[3] Kiss L (2019). Indirect foraminal decompression and improvement in the lumbar alignment after percutaneous cement discoplasty. Eur. Spine J..

[4] Techens C, Palanca M, Éltes PE, Lazáry Á, Cristofolini L (2020). Testing the impact of discoplasty on the biomechanics of the intervertebral disc with simulated degeneration: An in vitro study. Med. Eng. Phys..

[5] Eltes PE (2021). A novel three-dimensional volumetric method to measure indirect decompression after percutaneous cement discoplasty. J. Orthop. Transl..

[6] Tan JS, Uppuganti S (2012). Cumulative multiple freeze-thaw cycles and testing does not affect subsequent within-day variation in intervertebral flexibility of human cadaveric lumbosacral spine. Spine.

[7] Palanca M, Brugo T, Cristofolini L (2015). Use of Digital Image Correlationto investigate the biomechanics of the vertebra. J. Mech. Med. Biol..

[8] Bernardini F, Mittleman J, Rushmeier H, Silva C, Taubin G (1999). The ball-pivoting algorithm for surface reconstruction. IEEE Trans. Vis. Comput. Graph..

[9] Cignoni P, Rocchini C, Scopigno R (1998). Metro: Measuring error on simplified surfaces. Comput. Gr. Forum.

[10] Palanca M (2021). Type, size, and position of metastatic lesions explain the deformation of the vertebrae under complex loading conditions. Bone.

