# Supplementary material for: Assessment of foraminal decompression following discoplasty using a combination of ex vivo testing and numerical tools
Source: Sci Rep. 2023 Feb 25;13:3293. doi: 10.1038/s41598-023-27552-0 (PMC9968347; doi:10.1038/s41598-023-27552-0)
Supplement: Supplementary file 1 — Supplementary Information. [file 41598_2023_27552_MOESM1_ESM.pdf]

Supplementary Information to the paper
Assessment of foraminal decompression following discoplasty using a combination of *ex vivo* testing and numerical tools

Chloé Techens Ph.D[1,2], Ferenc Bereczki MD[2,3], Sara Montanari, Meng[1], Aron Lazary MD,PhD [2,4], Peter Endre Eltes MD, Ph.D[2,4#] and Luca Cristofolini, Ph.D [1#*]

Additional methodological details and results

S1 – Additional Materials and Methods

S1.1 – Cadaveric specimens

For this study, Functional Spinal Units (FSU) were extracted from 15 Caucasian lumbar spines (9 males/6 females) aged 35 to 86 y.o. Only specimens presenting intact endplates on computed tomography (CT) scan images were selected. The selection did not consider the degree of disc degeneration. The specimens were cleaned of the soft tissue keeping intact the anterior longitudinal, posterior longitudinal and interspinal ligaments. Each segment was aligned with the intervertebral disc horizontal. Both segment extremities were potted with acrylic cement.

S1.2 – Surgical procedure

PCD is a surgery recommended for advanced degeneration of the disc, when the nucleus pulposus is replaced by a vacuum phenomenon [2, 3]. As this specific state of degeneration is complicated to obtain in donor specimens, it was artificially created by manually emptying the disc to provide the anatomical vacuum characteristics needed for PCD using a substitutive method, thus providing a relevant and reproducible starting point. A rectangular incision as high as the disc and 5-8 mm wide was performed with a scalpel blade in the annulus fibrosus on the lateral side, preferably on the side showing irregularities (small osteophytes, wrinkled tissues). Lateral fenestration was chosen in consideration of the loading directions as it avoided damaging the tissue involved in flexion and extension biomechanics. The nucleus pulposus was extracted through the excision by a spine surgeon.

A highly radiopaque acrylic cement (Mendec Spine; Tecres, Sommacampagna, Italy, containing 30% BaSo4) was injected inside the disc through the incision until the cement would fill the cavity. The cement preparation was identical to clinical practice [3], mixing the components at room temperature, and waiting about 3-5 minutes to obtain the desired viscosity.

S1.3 – Biomechanical testing

Supplementary Table 1 – Donors' data and testing parameters for flexion and extension.

Specimen	*Sex-Age*	*Lumbar level*	*Offset (mm)*		*Axial displacement variation (mm)*		*Testing load (N)*	
			Flexion	*Extension*	*Flexion*	*Extension*	*Flexion*	*Extension*
01	M-68	T12-L1	12.3	24.5	-0.59	-1.35	402	
02	M-79	L2-L3	15.1	30.1	-	-0.61	-	387
03	M-53	L2-L3	13.1	26.3	-1.40	-0.73	402	
		L4-L5	13.6	27.2	-2.21	-0.15	402	
04	F-35	T12-L1	10.4	20.8	-0.37	-0.21	309	
		L2-L3	10.7	21.5	-3.20	-0.43	309	
		L4-L5	11.0	22.1	-	-0.34	-	309
05	F-68	T12-L1	9.1	18.2	-2.06	-0.84	396*	
06	M-59	L2-L3	10.9	21.8	0.49	-0.02	326*	
		L4-L5	11.6	23.2	0.59	-0.52	326*	140*
07	F-78	L1-L2	12.9	25.8	-0.46	-0.09	348	
		L3-L4	13.4	26.9	1.05	-0.69	348	
08	M-79	L1-L2	12.8	25.7	-2.02	-1.34	456	
		L3-L4	14.8	29.5	-1.22	-0.68	456	
09	F-86	L1-L2	13.6	27.2	-1.17	-0.56	265*	
		L3-L4	15.8	31.5	-0.92	-0.61	265*	
10	M-71	L1-L2	11.9	23.7	-1.98	-0.38	343	
		L3-L4	13.3	26.5	-	-0.40	-	343
11	M-68	L2-L3	12.6	25.3	-0.44	-0.11	319	
		L4-L5	13.2	26.3	-1.51	-0.01	319	
12	F-80	L3-L4	13.9	27.9	-0.63	-0.42	378	
13	M-64	L1-L2	12.8	25.6	-1.62	-0.09	417	
		L4-L5	14.9	29.8	-4.31	0.09	417	
14	M-73	L3-L4	16.6	33.1	-2.12	-0.57	515	
15	F-74	L1-L2	12.3	24.6	-1.65	-1.01	412	

*Reduced load to avoid damages

During each test, the 3-dimensional deformation distribution of the specimen surface were tracked using a Digital Image Correlation (DIC) system (Q400, Dantec Dynamics, Skovlunde, Denmark). For this purpose, a high-contrast speckle pattern was painted on both the vertebrae and the intervertebral disc using a methylene blue solution to stain the tissues and white water-based acrylic paint sprayed on top [5, 6]. Images were recorded at 15 Hz from the unloaded condition (reference frame) to the end of the 6th cycle. The DIC-correlated image corresponding to the 6th load peak was extracted from each test.

S1.4 – Post processing of DIC data

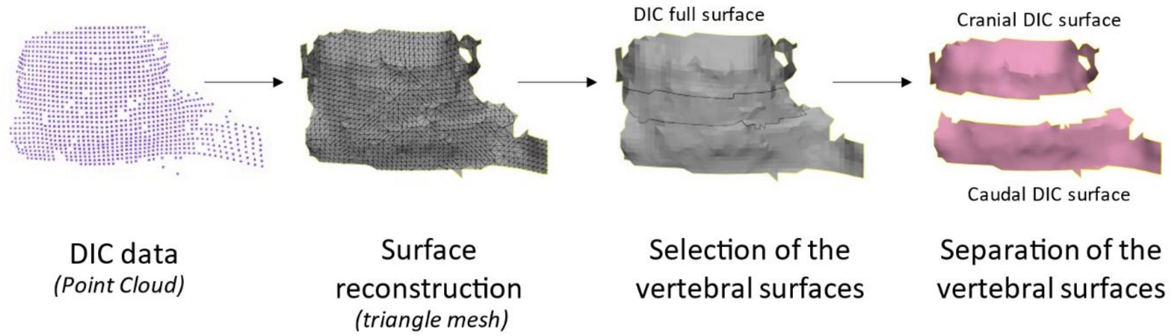

Fig. S1.1 – DIC data: from point clouds to identified vertebra surfaces.

S1.5 - Additional References

1. Tan JS, Uppuganti S (2012) Cumulative Multiple Freeze-Thaw Cycles and Testing Does Not Affect Subsequent Within-Day Variation in Intervertebral Flexibility of Human Cadaveric Lumbosacral Spine. SPINE 37:E1238–E1242. https://doi.org/10.1097/BRS.0b013e31826111a3

2. Varga PP, Jakab G, Bors IB, et al (2015) Experiences with PMMA cement as a stand-alone intervertebral spacer. Orthop 44:1–8. https://doi.org/10.1007/s00132-014-3060-1

3. Sola C, Camino Willhuber G, Kido G, et al (2018) Percutaneous cement discoplasty for the treatment of advanced degenerative disk disease in elderly patients. Eur Spine J. https://doi.org/10.1007/s00586-018-5547-7

4. Cottrell JM, van der Meulen MCH, Lane JM, Myers ER (2006) Assessing the Stiffness of Spinal Fusion in Animal Models. HSS J 2:12–18. https://doi.org/10.1007/s11420-005-5123-7

5. Lionello G, Sirieix C, Baleani M (2014) An effective procedure to create a speckle pattern on biological soft tissue for digital image correlation measurements. J Mech Behav Biomed Mater 39:1–8. https://doi.org/10.1016/j.jmbbm.2014.07.007

6. Palanca M, Tozzi G, Cristofolini L (2016) The use of digital image correlation in the biomechanical area: a review. Int Biomech 3:1–21. https://doi.org/10.1080/23335432.2015.1117395

S2 – Analysis of the parameters impacting the registration quality

S2.1 - Characterization of the DIC surfaces

As it was suspected that the quality of the overall registration could be affected by the quality of the DIC acquisition, the geometry of the correlated DIC surfaces was characterized in terms of dimension and unicity. The surface of the mesh was automatically measured by '3-matic'. The total contour of the mesh was computed by adding the length of the external and internal 'bad contours' reported by the software. Finally, in order to assess the specificity of the surface, its "roughness" was measured. Roughness usually characterizes very small asperities however here, the evaluated geometric irregularities of the DIC-acquired surfaces were larger (of the order 1-5mm), and these features were important for the registration. By measuring the roughness at this level, the asperities were identified on the surface and quantified by their height. For that, the point clouds of the DIC surfaces were primarily segmented into 80 000 points in CloudCompare v2.6.0 opensource software (R&D Institute EDF, Paris, France, https://www.danielgm.net/cc/). The roughness, corresponding to the distance between the point and the best fitted plane on the kernel, was computed using 'tool>other>roughness' tool. In order to target the main asperities of the DIC surface, kernel sizes in the range of the asperities were tested. A 3.0 mm kernel size was finally set, allowing the identification of the asperities characterizing the vertebra shape while excluding the noise asperities created by the remaining soft tissue. Because both the height and the number of asperities helped the manual registration, the roughness distribution histogram was extracted for each surface, and the mean and maximum of the distribution were computed. In addition, to quantify the number of high asperities (called density of asperities below), the number of mesh nodes exhibiting a local roughness >0.5 mm was derived using a Matlab script.

The relationships between the HD values and the DIC mesh surface, HD values and the roughness parameters were investigated with Spearman's rank correlation.

S2.2 – Correlation between DIC surfaces and registration precision

In order to have a more detailed assessment of the registration accuracy, the impact on the registration of the DIC mask characteristics were investigated. In particular, the total surface and the roughness of the masks were studied. The overall DIC mask surface area in the different specimens was at 440 ± 137 mm^2 (mean±s.d.). The maximum and mean roughness over all DIC masks were respectively 1.10 ± 0.36 mm and 0.11±0.02 mm. The mean density of asperities was 7973 nodes (range 0-64521). Weak but significant negative correlations (Fig. S2.1) were found between the DIC mask surface area and the mean HD values for O_1 (ρ= -0.419, $p<0.01$), the DIC mask surface and the mean HD values for O_2 (ρ= -0.358, $p<0.01$), the DIC mask surface and the maximum HD values for O_1 (ρ= -0.379, $p<0.01$), and the DIC mask surface and the maximum HD values for O_2 (ρ= -0.347, $p<0.01$). No significant correlation was found between the HD results and DIC mask roughness.

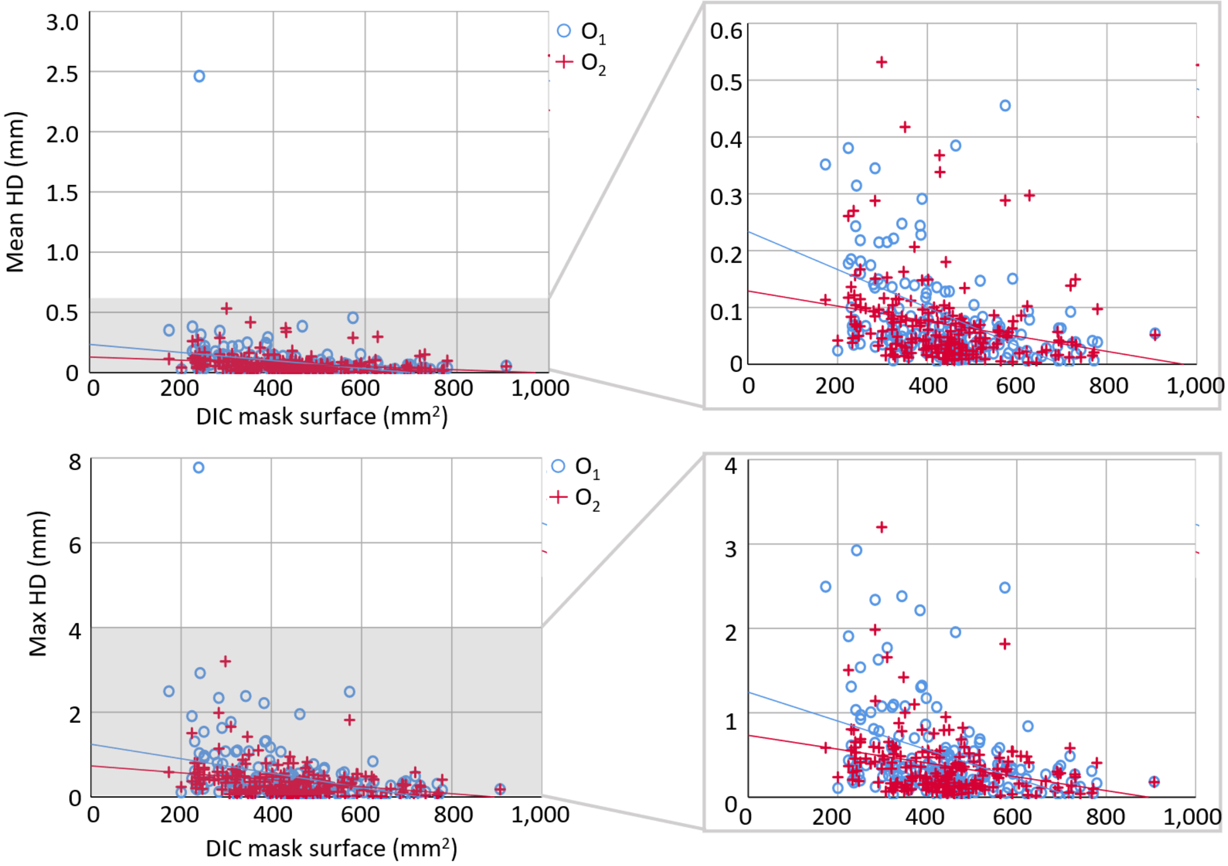

Fig. S2.1 – Correlations between the mean Hausdorff Distance (top), the maximum Hausdorff Distance (bottom) measured for operators O1 and O2, and the surface of each DIC mask. Linear regression is plotted for all data

S2.3 – Criteria on DIC surfaces

This analysis investigated the relationship between the DIC surface characteristics and the precision of registration. Among the studied parameters, only the area of the surface significantly impacted the registration repeatability. Unfortunately, this did not completely explain the outlier since 12 other specimens with lower surface area showed mean HD values in the overall range. Then, some other parameters should have probably also interfered and disrupted the registration results. Thus, if no strict criteria on the DIC surface area could be drawn, one should keep in mind that a narrower DIC surface induced more scattered HD values (s.d. for 25% narrowest surfaces: 0.36 mm for O_1 and 0.09 mm for O_2, s.d. for 25% largest surfaces: 0.07 mm for O_1 and 0.06 for O_2) and then a lower precision of the registration.

S3 – Additional Results

Supplementary Table 2 - Hausdorff Distance (HD) of the registered DIC masks. Measurements evaluated the accuracy of repeated registrations by the same operator (O_1T_1 vs O_1T_2, O_2T_1 vs O_2T_2) and the registration accuracy between two operators (O_1T_1 vs O_2T_1, O_1T_2 vs O_2T_2). Results are presented as the mean of HDs over all the DIC masks (s.d.).

		**HD (mm)**			
	**Compared surfaces**	**Min**	**Mean**	**Max**	**RMS**
Intra-operator	O_1T_1 vs O_1T_2	0.00 (0.00)	0.10 (0.23)	0.52 (0.79)	0.13 (0.28)
	O_2T_1 vs O_2T_2	0.00 (0.01)	0.07 (0.07)	0.37 (0.38)	0.10 (0.10)
Inter-operator	O_1T_1 vs O_2T_1	0.01 (0.03)	0.18 (0.25)	0.63 (0.87)	0.21 (0.29)
	O_1T_2 vs O_2T_2	0.01 (0.04)	0.15 (0.18)	0.53 (0.65)	0.18 (0.22)

Legend: Min=minimum, Max=maximum, RMS=root mean square

Supplementary Table 3 - Volumes and changes based on the measurements of O_1 and O_2 at T_1 and T_2 ($O_1T_1+O_1T_2+O_2T_1+O_2T_2/4$).
Mean and standard deviation (s.d.) are reported for the 15 specimens.

		Flexion					*Extension*				
Specimen ID	*Spine level*	*Cylinder radius (mm)*	*Mean $V_{nucleotomy}$ (mm³)*	*Mean $V_{discoplasty}$ (mm³)*	*Mean ΔV (mm³)*	*s.d. ΔV (mm³)*	*Cylinder radius (mm)*	*Mean $V_{nucleotomy}$ (mm³)*	*Mean $V_{discoplasty}$ (mm³)*	*Mean ΔV (mm³)*	*s.d. ΔV (mm³)*
01	T12-L1	11	28524	29573	1049	326	11	28379	28869	490	64
02	L2-L3	-	-	-	-	-	13	35869	37013	1143	216
03	L2-L3	13	41234	41665	431	40	13	38748	39218	470	87
03	L4-L5	12	31432	33222	1789	143	12	29608	31319	1711	160
04	T12-L1	12	34993	35351	358	216	12	34584	35655	1071	393
04	L2-L3	12	36287	34688	-1599	265	12	33983	34301	318	186
04	L4-L5	-	-	-	-	-	12	32143	32662	519	839
05	T12-L1	12	35750	36362	613	236	12	35088	35498	411	102
06	L2-L3	11	30383	30585	202	186	11	28824	29231	407	243
06	L4-L5	10	24146	24702	556	159	11	22459	23880	1422	356
07	L1-L2	11	29112	29544	432	222	11	28316	28260	-55	183
07	L3-L4	10	22830	25700	2870	955	10	20682	23095	2413	605
08	L1-L2	11	28524	28576	52	256	11	26014	29534	3521	378
08	L3-L4	15	26456	31604	2512	3049	15	29092	30950	4494	1110
09	L1-L2	11	28898	29391	493	552	11	28358	29629	1272	379
09	L3-L4	-	-	-	-	-	13	37393	39778	2385	2035
10	L1-L2	12	32750	33022	272	359	12	31581	32383	802	238
10	L3-L4	-	-	-	-	-	12	34431	35136	705	273
11	L2-L3	12	35357	35516	159	172	11	30962	31070	109	115
11	L4-L5	12	32079	33036	956	363	11	26670	28715	2045	358
12	L3-L4	11	27565	27410	-155	133	11	26406	26505	99	225
13	L1-L2	11	29931	30684	753	150	11	29074	29789	715	161
13	L4-L5	12	29180	33896	4716	1784	12	30665	32726	2061	298
14	L3-L4	12	32750	33835	1085	388	12	30464	31340	876	333
15	L1-L2	12	36997	36996	-2	179	12	35222	35946	724	179
**Mean**					**835**					**1205**	
**s.d.**					**1289**					**1106**	

Supplementary Table 4 - Volumetric measurements done by operator one (O_1), at two times (T_1, T_2). The main result is $\Delta V = V_{discoplasty} - V_{nucleotomy}$ the volumetric change of the foramen induced by discoplasty.

		Flexion						Extension					
		O_1T_1			O_1T_2			O_1T_1			O_1T_2		
Specimen ID	Spine level	$V_{nucleotomy}$ (mm³)	$V_{discoplasty}$ (mm³)	ΔV (mm³)	$V_{nucleotomy}$ (mm³)	$V_{discoplasty}$ (mm³)	ΔV (mm³)	$V_{nucleotomy}$ (mm³)	$V_{discoplasty}$ (mm³)	ΔV (mm³)	$V_{nucleotomy}$ (mm³)	$V_{discoplasty}$ (mm³)	ΔV (mm³)
01	T12-L1	28504	29863	1359	28405	29703	1298	28457	29023	566	28688	29205	517
02	L2-L3	-	-	-	-	-	-	35724	36632	907	35354	36779	1425
03	L2-L3	41312	41774	462	41240	41627	387	39025	39380	355	39078	39629	551
03	L4-L5	31970	33614	1644	31358	33230	1872	29884	31628	1744	29728	31433	1705
04	T12-L1	35301	35586	285	35224	35343	119	34562	35531	969	34560	36210	1650
04	L2-L3	36256	35041	-1215	36455	34775	-1680	34037	34269	232	34030	34205	175
04	L4-L5	-	-	-	-	-	-	33043	33027	-16	32661	32340	-321
05	T12-L1	35235	36171	936	35701	36270	569	34889	35435	546	34879	35295	416
06	L2-L3	30536	30599	63	30371	30770	399	28641	29124	483	28308	28877	569
06	L4-L5	22497	23157	660	22576	23256	680	20836	22283	1447	20359	22273	1914
07	L1-L2	28873	29601	728	29276	29512	236	28490	28501	11	28509	28653	144
07	L3-L4	20188	22328	2140	22514	24621	2107	18619	21103	2484	17505	20736	3231
08	L1-L2	28388	28801	413	28737	28620	-117	26586	29735	3149	25777	29636	3859
08	L3-L4	25795	23783	-2012	23791	28274	4483	22603	27897	5294	22774	25658	2884
09	L1-L2	28792	30097	1305	28895	29029	134	28361	29185	824	28310	29428	1118
09	L3-L4	-	-	-	-	-	-	39407	43374	3967	39129	42791	3662
10	L1-L2	30604	31207	603	30448	30365	-83	28921	30079	1158	29385	30095	710
10	L3-L4	-	-	-	-	-	-	34761	35513	752	34723	35127	404
11	L2-L3	35317	35376	59	35222	35407	185	28445	28696	251	28608	28578	-30
11	L4-L5	31761	32543	782	32205	32738	533	24751	26304	1553	24230	26531	2301
12	L3-L4	27576	27565	-11	27777	27515	-262	26558	26605	47	26576	26385	-191
13	L1-L2	29930	30645	715	29746	30707	961	29190	29788	598	29142	29697	555
13	L4-L5	27969	33972	6003	27061	33551	6490	30532	32686	2154	30238	32262	2024
14	L3-L4	32759	33865	1106	33169	33714	545	30438	31257	819	31033	31471	438
15	L1-L2	37114	37309	195	36928	37031	103	35418	35953	535	35361	35999	638
**Mean**				**772**			**903**			**1233**			**1214**
**s.d.**				**1499**			**1737**			**1304**			**1208**

Supplementary Table 5 - Volumetric measurements done by operator two (O_2), at two times (T_1, T_2). The main result is $\Delta V = V_{discoplasty} - V_{nucleotomy}$ the volumetric change of the foramen induced by discoplasty.

		Flexion						Extension					
		O_2T_1			O_2T_2			O_2T_1			O_2T_2		
**Specimen ID**	**Spine level**	$V_{nucleotomy}$ (mm^3)	$V_{discoplasty}$ (mm^3)	ΔV (mm^3)	$V_{nucleotomy}$ (mm^3)	$V_{discoplasty}$ (mm^3)	ΔV (mm^3)	$V_{nucleotomy}$ (mm^3)	$V_{discoplasty}$ (mm^3)	ΔV (mm^3)	$V_{nucleotomy}$ (mm^3)	$V_{discoplasty}$ (mm^3)	ΔV (mm^3)
01	T12-L1	28743	29460	717	28443	29266	823	28292	28718	426	28078	28529	451
02	L2-L3	-	-	-	-	-	-	36058	37227	1169	36341	37413	1072
03	L2-L3	41079	41547	468	41306	41713	407	38420	38873	453	38470	38990	520
03	L4-L5	31251	32946	1695	31150	33096	1946	29259	30762	1503	29560	31451	1891
04	T12-L1	34762	35397	635	34684	35076	392	34248	35040	792	34965	35838	873
04	L2-L3	36243	34568	-1675	36192	34367	-1825	33936	34526	590	33928	34202	274
04	L4-L5	-	-	-	-	-	-	31305	32807	1502	31563	32475	912
05	T12-L1	36198	36568	370	35864	36440	576	35370	35749	379	35212	35513	301
06	L2-L3	30207	30529	322	30416	30441	25	29285	29815	530	29062	29108	46
06	L4-L5	25322	25872	550	26188	26522	334	23942	25146	1204	24697	25819	1122
07	L1-L2	29254	29729	475	29043	29333	290	28322	28237	-85	27941	27650	-291
07	L3-L4	24447	27561	3114	24169	28289	4120	23137	25225	2088	23465	25314	1849
08	L1-L2	28414	28469	55	28557	28414	-143	25641	29475	3834	26050	29291	3241
08	L3-L4	33557	36963	3406	33225	37394	4169	29945	34579	4634	30502	35665	5163
09	L1-L2	29191	29347	156	28715	29091	376	28306	29994	1688	28453	29909	1456
09	L3-L4	-	-	-	-	-	-	36201	35706	-495	34835	37240	2405
10	L1-L2	34916	34926	10	35032	35591	559	33781	34450	669	34237	34908	671
10	L3-L4	-	-	-	-	-	-	34014	35068	1054	34226	34835	609
11	L2-L3	35344	35734	390	35545	35546	1	33318	33425	107	33475	33581	106
11	L4-L5	32407	33726	1319	31944	33135	1191	28807	31128	2321	28893	30898	2005
12	L3-L4	27479	27205	-274	27426	27355	-71	26376	26587	211	26114	26442	328
13	L1-L2	29984	30716	732	30064	30667	603	29015	29872	857	28949	29800	851
13	L4-L5	30625	33995	3370	31063	34065	3002	31180	32857	1677	30710	33100	2390
14	L3-L4	32435	33892	1457	32637	33867	1230	30331	31368	1037	30053	31263	1210
15	L1-L2	36993	36865	-128	36954	36777	-177	35004	35953	949	35106	35880	774
**Mean**				**817**			**849**			**1164**			**1209**
**s.d.**				**1243**			**1426**			**1144**			**1185**

Supplementary Table 6 – Mean and Standard Deviation of the foramen volumetric change depending on the treated spine level. Results were reported for flexion and extension.

	Flexion		Extension	
	**Mean (mm^3)**	**s.d. (mm^3)**	**Mean (mm^3)**	**s.d (mm^3)**
**T12-L1**	673	350	657	361
**L1-L2**	333	295	1163	1231
**L2-L3**	-202	1102	489	390
**L3-L4**	1578	876	1829	1607
**L4-L5**	2004	1975	1552	635
